# Effects of Olfactory Valence on the Neural and Behavioral Dynamics of Approach-Avoidance: An EEG Study

**DOI:** 10.3390/brainsci15101041

**Published:** 2025-09-25

**Authors:** Yang Yang, Xiaochun Wang

**Affiliations:** School of Psychology, Shanghai University of Sport, Shanghai 200438, China; y_yang1004@163.com

**Keywords:** olfactory valence, approach-avoidance behavior, cognitive control, motivation, attention

## Abstract

**Background/Objectives**: Approach-avoidance behavior is critical for adaptive behavior. The neural basis of these behaviors has been investigated extensively, but the effect of odor valence is unclear. This study tested how positive, negative, and neutral odors affect behavior and event-related potentials (ERPs) in the approach-avoidance task (AAT). **Methods**: Thirty-two healthy participants performed an AAT. We measured reaction time, accuracy, and ERP components (P1, N1, N2, P3) to understand the process of motivational processing over time. **Results**: Participants responded faster and more accurately when the direction and target type were congruent under all odor conditions. Odors did not change this core consistent pattern. In contrast, ERP results revealed stage-specific modulations. P1 and N1 components reflected odor-related changes in early sensory processing. The N2 effect present under the air condition was largely absent under positive and negative odors. This suggests reduced conflict monitoring. P3 amplitudes were consistently larger for avoidance than for approach responses, regardless of odor valence. **Conclusions**: The results demonstrate that odor valence reorganized the neural dynamics of the AAT without changing behavioral performance. This finding shows that olfactory valence modulates attention and control mechanisms and plays a unique role in regulating human motivation.

## 1. Introduction

Approach and avoidance behaviors are fundamental to survival, enabling organisms to obtain rewards and evade threats in dynamic environments [1,2]. These behaviors are shaped not only by internal states such as emotion and arousal, but also by external cues that signal affective or motivational relevance. Among these cues, olfaction holds a special status due to its unique neuroanatomical pathways and evolutionary role in detecting food, danger, and social signals. In contrast to other sensory modalities, olfactory inputs bypass the thalamus and project directly to limbic structures, enabling rapid responses. This direct neural pathway positions olfaction as a powerful and evolutionarily conserved modulator of motivational action. Olfactory processing is largely automatic and often occurs without conscious attention, as it is intrinsically linked to the act of breathing [3].

Olfactory cues influence a wide range of cognitive and affective processes. Emotionally salient odors can modulate visual attention, temporal perception, and even social evaluation, while evoking stronger emotions and autobiographical memories. The valence of a stimulus, whether it is positive or negative, plays an important role in shaping motivational tendencies. Pleasant odors can enhance positive affect and promote relaxation, whereas unpleasant odors heighten vigilance and bias perception toward threat [4,5]. The impact of odor valence extends beyond simple perception, directly impacting perceptual decision-making and motor behavior [6,7]. For example, unpleasant odors can rapidly activate brain regions related to threat and aggression, ultimately leading to faster motor avoidance responses [6,8]. Despite this strong link between olfaction and motivational states, the neurocognitive mechanisms underlying this dynamic influence on automatic approach-avoidance tendencies are not yet fully understood.

From a neurocognitive perspective, approach–avoidance behavior reflects the interaction between rapid, affectively driven processes and slower, controlled processes. The dual-systems framework proposes that emotionally valence cues can automatically bias motor tendencies, which may then be overridden or adjusted by executive control mechanisms [9,10,11,12]. Event-related potential (ERP) studies have provided a robust temporal framework for understanding this process. Early components like P1 and N1 reflect sensory-attentional orienting; the N2 indexes conflict monitoring; and the P3 reflects the integration of affective and task-related information during decision-making [13,14,15]. This refined temporal cascade provides an ideal basis for studying how odor valence, as the primary background signal, selectively modulates actions at different stages.

The Approach–Avoidance Task (AAT) is a validated paradigm for probing these mechanisms. It reliably produces a consistent effect, where responses are faster when movement direction matches stimulus valence [16]. A great deal of research has been dedicated to understanding the effects of visual and auditory cues in the AAT [17,18,19]. However, there is still much to be learned about the potential of olfactory valence. This is a crucial omission, as odors are constant features of our naturalistic, multi-sensory environments and can influence behavior. Investigating how these biologically salient and ancient signals interact with the motor component of the AAT is vital for a complete understanding of affect–motivation integration.

The present study addressed this gap by examining how odor valence (positive, negative, and air) influences behavioral performance and the underlying neural dynamics during an AAT. We used a 3 (Odor Valence: positive, negative, air) × 2 (Response Direction: approach, avoidance) × 2 (Target Type: neutral, dangerous) within-subject design, with EEG recording to capture the temporal cascade of motivational action. Based on prior literature, we hypothesized that:

- Early sensory-attentional components (P1/N1) would be modulated by odor valence, indicating that olfactory cues can influence the perceptual processing of motivationally relevant stimuli;
- The conflict-related N2 component would vary with odor valence, consistent with the notion that odors may alter general control demands during response selection;
- P3 amplitude may reflect late-stage processing of response tendencies under odor conditions.

This study aims to provide a temporally precise account of how odor valence reorganizes the neurocognitive stages of motivation. Our findings extend affect motivation theories to olfaction and illustrate how olfactory cues influence neural stages of motivated action.

## 2. Materials and Methods

### 2.1. Participants

We calculated the required sample size using G*Power software (version 3.1.9.2). The power analysis was based on a two-way repeated measures ANOVA, with an effect size of 0.25, a statistical power of 0.8, and a significance level of 0.05. The results indicated that a minimum of 30 participants were needed. A total of 34 participants were recruited for the study. Two participants dropped out during the experiment, so 32 participants’ data were included in the analysis. The final sample consisted of 15 males (22.53 ± 2.45 years) and 17 females (22.30 ± 1.86 years).

All participants were students at Shanghai University of Sport. Inclusion criteria included normal or corrected-to-normal vision, right-handedness, and self-reported normal olfactory function, with no history of olfactory disorders such as rhinitis. Exclusion criteria included any history of mental or neurological disorders or current medication use. Participants were also required to refrain from eating, smoking, or using perfume for at least two hours prior to the experiment. They read and signed an informed consent form in accordance with the principles outlined in the Declaration of Helsinki. This research was approved by the ethics committee of Shanghai University of Sport (102772023RT136).

### 2.2. Equipment and Stimuli

The experimental procedure was programmed using E-Prime 3.0. EEG data were recorded using BrainAmp Standard system (Brain Products GmbH, Gilching, Germany) and a 64-channel electrode cap based on the international 10–20 system. The sampling rate was 1000 Hz. Electrodes were also placed approximately 1 cm below the right eyelid to record EOG and exclude eye movement artifacts. Before the experiment, electrode impedances were kept below 5 kΩ. Participants were instructed to remain motionless and avoid blinking or moving during the experiment to minimize EEG signal interference.

Olfactory stimuli were delivered by an Olfactometer (ETT, College Station, TX, USA), which precisely controls gas flow through each channel. Its initiation, termination, and channel switching are seamlessly controlled via standard programming platforms like E-Prime 3.0 and MATLAB.

The target stimuli were selected from previous studies [20,21,22,23]. A total of 32 pictures were chosen, including 16 neutral and 16 dangerous images. In order to screen these target stimuli, we invited 24 students (14 females and 10 males) to evaluate the familiarity, risk and arousal of the pictures using a 7-point Likert scale. The final stimuli consist of 24 pictures, including 12 neutral targets (e.g., almonds, lychees, mineral water bottles, etc.) and 12 dangerous targets (e.g., syringes, razor blades, fishhooks, etc.). These targets were selected based on the ratings and used as target pictures in the AAT. The mean familiarity score for these targets was 6.79 ± 0.41. A one-way ANOVA showed no significant differences between neutral and dangerous images in familiarity and arousal ratings. However, a significant difference was observed in danger ratings (F(1, 46) = 859.375, *p* < 0.001, η_p_^2^ = 0.949, Table 1). All target pictures were converted to grayscale and placed on a white background. In the formal experiment, the presentation of these target pictures was randomized.

The olfactory stimuli were pre-evaluated before the formal experiment. 13 students rated the familiarity, danger, and intensity of nine candidate odors on a 7-point Likert scale. The odor with the highest pleasantness score was selected as the positive valence odor, and the one with the lowest pleasantness score was selected as the negative valence odor. The selected olfactory stimuli were a pleasant rose scent (phenylethyl alcohol, Sigma-Aldrich, St. Louis, MO, USA) and an unpleasant fecal odor (3-methylindole, Sigma-Aldrich). Phenylethyl alcohol was diluted in propylene glycol to a final concentration of 0.5% *v*/*v*, and 3-methylindole was diluted to a final concentration of 0.2% *v*/*v* [24]. To ensure that the pleasant and unpleasant odors were of comparable intensity, a group of four other students conducted a paired comparison. The final concentration of the odorants was determined by their consensus, ensuring that the intensities were approximately equivalent.

To prevent a decline in stimulus intensity due to evaporation, which can occur in conventional olfactory tests [25], volatile odors were delivered continuously to participants’ heads via an olfactometer’s compression pump and fixed tubing. Furthermore, to mitigate olfactory adaptation, which can reduce sensitivity during prolonged exposure [26,27,28], an intermittent delivery method was used. Odors were released for six seconds, followed by a 12 s pause. This diffusion method’s effectiveness has been confirmed in previous olfactory studies [29,30]. During the experiment, a constant air stream of 0.5 L/min was maintained. Odor stimuli were delivered through plastic tubing at a flow rate of 3 L/min, which was released into the room to create the scented environment [31].

### 2.3. Procedure

Participants completed an AAT under three odor conditions (positive, negative, and air) using a fully randomized within-subjects design. Each block contained 80 trials, followed by a mandatory 10 min break during which participants could drink purified water. To prevent odor carryover, the olfactometer was flushed during these breaks. Prior to and immediately after each odor block, participants were briefly exposed to the respective odor stimulus and asked to verbally indicate whether it was perceived as positive, negative, or air. This brief manipulation check was implemented to confirm that participants could perceptually discriminate against the odor valence during the experiment.

**Figure 1:**
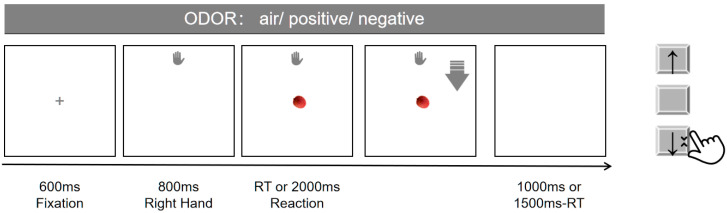
AAT procedure. A picture of the participant’s right hand appeared at the top or bottom of the screen, followed by a central target image. Participants responded by approaching or avoiding the target as instructed. Arrows indicate the required response direction.

Before the experiment, a photograph of each participant’s right hand was taken for use as a stimulus. The formal experimental procedure is shown in Figure 1. Each trial began with a fixation point presented for 600 ms. Next, the participant’s hand image appeared at the top or bottom of the screen for 800 ms, followed by the target image in the center. Participants were required to respond as quickly and accurately as possible by approaching or avoiding the target according to task instructions. Participants performed the task using a vertically arranged 3-key keyboard. During the preparation phase, the right index finger rested on the middle key. The upper and lower keys were assigned to move the right hand upward and move the right hand downward. Half of the trials required approaching neutral targets and avoiding dangerous targets, while the other half required the reverse.

### 2.4. Data Collection and Analysis

In the formal experiment, both behavioral and EEG data were recorded. Response times and accuracy rates for the AAT were automatically collected using E-Prime 3.0. Trials with incorrect responses or data points exceeding the mean ± 2 standard deviations were excluded from analysis. On average, 3.1%, 3.9%, and 4.8% of trials were, respectively, removed in the positive, negative, and air odor conditions. Behavioral and EEG analyses were based on each participant’s mean values for accuracy, response times, and ERP amplitudes to ensure data independence.

Behavioral and EEG measures were analyzed using a 3 (odor) × 2 (response direction) × 2 (target type) repeated-measures ANOVA in SPSS 26.0 with α = 0.05 (two-tailed). Mauchly’s test was applied to assess the sphericity assumption. If the sphericity assumption was violated, degrees of freedom were adjusted using the Greenhouse-Geisser correction to provide more conservative F- and *p*-values. Normality of residuals was evaluated visually and confirmed with Shapiro–Wilk tests on ANOVA residuals (all *p* > 0.05). Post hoc pairwise comparisons following significant effects were corrected using the Bonferroni method.

EEG data were processed in EEGLAB (MATLAB R2022a). EOG channels were excluded from subsequent analyses. The signals were band-pass filtered between 0.1 and 25 Hz and notch filtered at 48–52 Hz to suppress 50 Hz mains interference. Data were downsampled to 500 Hz, bad channels were interpolated using spherical splines, and the data were re-referenced to the average of all electrodes. To reduce artifacts, participants were instructed to minimize head and body movements, facial expressions, swallowing, and eye blinks. Independent component analysis (ICA) using the runica algorithm was applied to identify and remove components associated with ocular or muscle artifacts, including EMG, ECG, and EOG activity. Following ICA, data were segmented into epochs from −200 to 1000 ms relative to target onset and baseline-corrected using the −200 to 0 ms pre-stimulus interval. Epochs containing incorrect responses or voltage exceeding ±100 µV were rejected. Electrode sites and time windows for each ERP component were selected based on previous literature [32,33,34] and confirmed by visual inspection of grand-averaged waveforms.

Descriptive statistics are reported as means ± standard deviations (M ± SD), while figures display mean values with standard error bars to represent variability for inferential purposes.

## 3. Results

### 3.1. Behavioral Results

#### 3.1.1. Reaction Times (Figure 2, Left Panels)

The analysis revealed a significant main effect of odor (F(2, 62) = 7.756, *p* = 0.002, η_p_^2^ = 0.200) and a significant interaction between response direction and target type (F(1,31) = 45.474, *p* < 0.001, η_p_^2^ = 0.595). The main effects of target type (F(1, 31) = 2.477, *p* = 0.126, η_p_^2^ = 0.074) and direction, F(1, 31) = 2.395, *p* = 0.132, η_p_^2^ = 0.072, were not significant. Likewise, the interactions of odor × target type, F(2, 62) = 1.414, *p* = 0.251, η_p_^2^ = 0.044, and odor × response direction (F(2, 62) = 1.683, *p* = 0.194, η_p_^2^ = 0.051) as well as the three-way interaction (F(2, 62) = 0.544, *p* = 0.562, η_p_^2^ = 0.017) were not significant.

**Figure 2:**
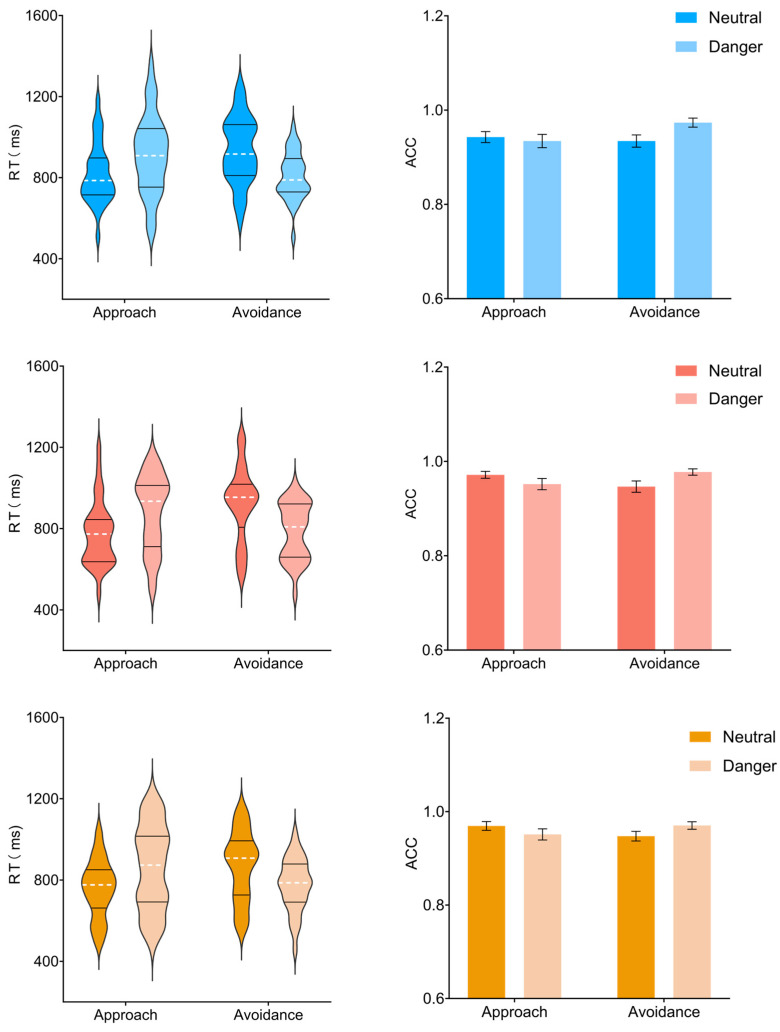
Reaction times (RTs, **left panels**) and accuracy rates (ACC, **right panels**) under three odor conditions. Data are shown separately for air (**top row**), positive (**middle row**), and negative (**bottom row**) odor blocks. Error bars represent standard errors of the mean (SE). Black horizontal lines indicate the first and third quartiles (Q1 and Q3) of the data distribution. The white dashed line represents the mean RT.

Post hoc comparisons for the main effect of odor showed that RTs in the air condition (868.99 ± 24.71 ms) were significantly slower than in the negative odor condition (816.82 ± 26.22 ms, *p* = 0.003, 95% CI [15.713, 88.630]). The difference between the air and positive odor conditions (840.57 ± 27.07 ms, *p* = 0.183) and between the positive and negative odor conditions (*p* = 0.085) did not reach significance.

Simple effect analyses of the response direction × target type interaction revealed a robust approach–avoidance pattern. When the target stimulus was neutral, approach responses (784.89 ± 24.12 ms) were significantly faster than avoidance responses (908.93 ± 28.26 ms, *p* < 0.001, 95% CI [−160.447 −87.619]). In contrast, when the target stimulus was dangerous, approach responses (884.83 ± 32.86 ms) were significantly slower than avoidance responses (789.84 ± 20.04 ms, *p* < 0.001, 95% CI [54.989, 134.996]). Furthermore, within the approach direction, RTs were faster for neutral than dangerous targets (*p* < 0.001, 95% CI [−135.426, −64.450]), whereas within the avoidance direction, RTs were slower for neutral than dangerous targets (*p* < 0.001, 95% CI [83.838, 154.336]).

#### 3.1.2. Accuracy (Figure 2, Right Panels)

The three-way ANOVA on accuracy revealed a significant interaction between target type and response direction (F(1, 31) = 9.683, *p* = 0.004, η_p_^2^ = 0.238). Main effects of odor (F(2, 62) = 3.050, *p* = 0.063, η_p_^2^ = 0.090), target type (F(1, 31) = 2.382, *p* = 0.133, η_p_^2^ = 0.071), and response direction (F(1, 31) = 1.065, *p* = 0.310, η_p_^2^ = 0.033), as well as interactions between odor and target (F(2, 62) = 0.870, *p* = 0.415, η_p_^2^ = 0.027) and odor and direction (F(2, 62) = 1.326, *p* = 0.272, η_p_^2^ = 0.041), and the three-way interaction (F(2, 62) = 0.085, *p* = 0.886, η_p_^2^ = 0.003), were not significant.

Simple effects analyses of the target × direction interaction showed that for neutral targets, approach responses (0.96 ± 0.01) were more accurate than avoidance responses (0.94 ± 0.01, *p* = 0.048, 95% CI [0, 0.037]). For dangerous targets, approach responses (0.95 ± 0.01) were less accurate than avoidance responses (0.97 ± 0.01, *p* = 0.002, 95% CI [−0.044, −0.011]). When the response direction was approaching, accuracy did not differ significantly between neutral and dangerous targets (*p* = 0.106, 95% CI [−0.004, 0.036]), whereas for avoidance responses, accuracy was lower for neutral compared to dangerous targets (*p* < 0.001, 95% CI [−0.045, −0.014]).

### 3.2. ERP Results

#### 3.2.1. N1 Component (Figure 3)

Time window: 190–230 ms

A three-way repeated-measures ANOVA on N1 amplitude revealed significant main effects of odor valence (F(2, 62) = 4.632, *p* = 0.014, η_p_^2^ = 0.138) and response direction (F(1, 31) = 37.714, *p* < 0.001, η_p_^2^ = 0.565), as well as a significant interaction between response direction and target type (F(1, 31) = 5.292, *p* = 0.029, η_p_^2^ = 0.154). The main effect of target type (F(1, 31) = 2.598, *p* = 0.118, η_p_^2^ = 0.082), the interactions of odor × response direction (F(2, 62) = 0.567, *p* = 0.556, η_p_^2^ = 0.019) and odor × target type (F(2, 62) = 0.522, *p* = 0.554, η_p_^2^ = 0.018), and the three-way interaction (F(2, 62) = 1.631, *p* = 0.205, η_p_^2^ = 0.053) were not significant.

Post hoc pairwise comparisons indicated that, regardless of response direction and target type, N1 amplitude in the air condition (−1.37 ± 0.53 µV) was significantly more negative than in the positive odor condition (−0.61 ± 0.51 µV, *p* = 0.033, 95% CI [−1.461, −0.047]) and marginally more negative than in the negative odor condition (−0.56 ± 0.49 µV, *p* = 0.052, 95% CI [−1.619, 0.006]), with no significant difference between the positive and negative odor conditions (*p* = 0.895).

For response direction, avoidance responses elicited significantly more negative N1 amplitudes (−1.40 ± 0.48 µV) than approach responses (−0.29 ± 0.49 µV, *p* < 0.001, 95% CI [0.742, 1.483]). Simple effect analyses of the response direction × target type interaction revealed that for neutral targets, N1 amplitude for avoidance responses (−1.81 ± 0.48 µV) was significantly more negative than for approach responses (−0.17 ± 0.48 µV, *p* < 0.001, 95% CI [1.011, 2.266]); for dangerous targets, N1 amplitude for avoidance responses (−0.99 ± 0.54 µV) was also significantly more negative than for approach responses (−0.40 ± 0.53 µV, *p* = 0.042, 95% CI [0.021, 1.150]). When examining the effect of target type within each response direction, no significant difference was observed between approach responses to neutral and dangerous targets (*p* = 0.360), whereas for avoidance responses, N1 amplitude was significantly more negative for neutral targets than for dangerous targets (*p* = 0.020, 95% CI [−1.503, −0.142]).

**Figure 3:**
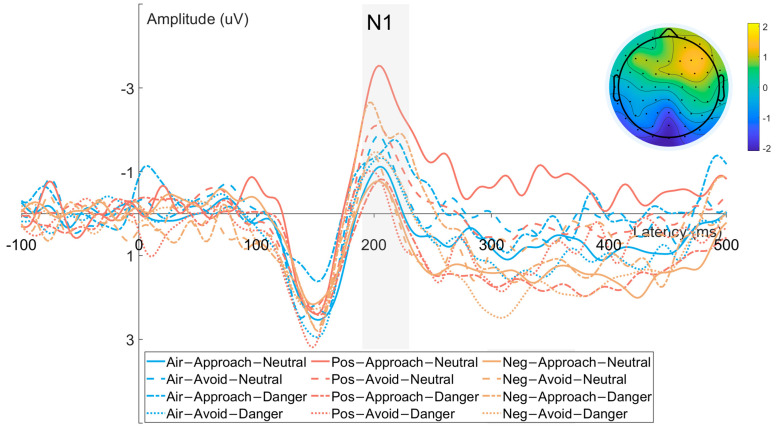
Waveforms and scalp topographies of the N1 component recorded from occipital electrodes (O1, O2, Oz).

#### 3.2.2. N2 Component (Figure 4)

Time window: 280–350 ms

Electrodes: Fz, Cz

The amplitude of the N2 component revealed significant main effects of odor valence (F(2, 62) = 6.790, *p* = 0.004, η_p_^2^ = 0.190) and response direction (F(1, 31) = 6.304, *p* = 0.018, η_p_^2^ = 0.179). However, the main effect of target type (F(1, 31) = 1.128, *p* = 0.297, η_p_^2^ = 0.037), the interaction between odor valence and response direction (F(2, 62) = 0.633, *p* = 0.501, η_p_^2^ = 0.021), the interaction between odor valence and target type (F(2, 62) = 0.236, *p* = 0.780, η_p_^2^ = 0.008), the interaction between response direction and target type (F(1, 31) = 2.883, *p* = 0.100, η_p_^2^ = 0.090), and the three-way interaction among odor valence, target type, and response direction (F(2, 62) = 1.402, *p* = 0.254, η_p_^2^ = 0.046) were all non-significant.

Post hoc comparisons indicated that N2 amplitudes were significantly more negative in both the positive odor condition (−2.57 ± 0.37 μV, *p* = 0.046, 95% CI [0.008, 1.102]) and negative odor condition (−2.90 ± 0.34 μV, *p* = 0.016, 95% CI [0.137, 1.620]) relative to the air condition (−2.02 ± 0.36 μV), whereas no significant difference was observed between the positive and negative odor conditions (*p* = 0.387). Regarding response direction, N2 amplitudes were significantly more negative for approach trials (−2.68 ± 0.31 μV) than for avoidance trials (−2.30 ± 0.37 μV, *p* = 0.018, 95% CI [−0.695, −0.071]).

**Figure 4:**
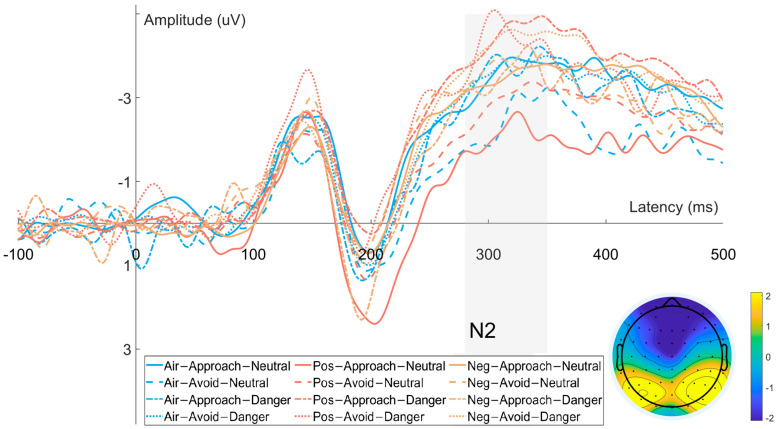
Waveforms and scalp topographies of the N2 component recorded from frontal–central electrodes (Fz, Cz).

#### 3.2.3. P1 Component (Figure 5)

Time window: 130–160 ms

The ANOVA results showed that there were no significant main effects of odor valence (F(2, 62) = 0.612, *p* = 0.518, η_p_^2^ = 0.021), response direction (F(1, 31) = 0.591, *p* = 0.448, η_p_^2^ = 0.020), or target type (F(1, 31) = 1.193, *p* = 0.284, η_p_^2^ = 0.004) on P1 amplitude. Additionally, none of the two-way interactions—odor × response direction (F(2, 62) = 0.346, *p* = 0.676, η_p_^2^ = 0.012), odor × target type (F(2, 62) = 2.023, *p* = 0.144, η_p_^2^ = 0.065), or response direction × target type (F(1, 31) = 0.344, *p* = 0.562, η_p_^2^ = 0.012) were significant. The three-way interaction among odor, response direction, and target type was also not significant (F(2, 62) = 2.040, *p* = 0.142, η_p_^2^ = 0.066).

**Figure 5:**
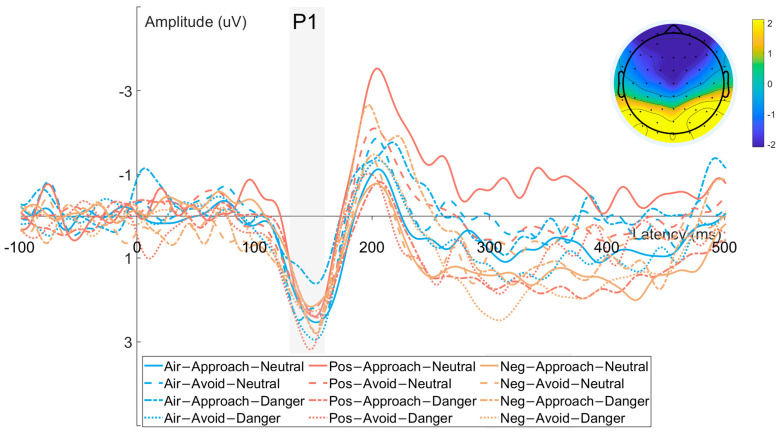
Waveforms and scalp topographies of the P1 component recorded from occipital electrodes (O1, O2, Oz).

#### 3.2.4. P3 Component (Figure 6)

Time window: 300–400 ms

Electrodes: Pz

The ANOVA results showed a significant main effect of response direction (F(1, 31) = 59.419, *p* < 0.001, η_p_^2^ = 0.672) on P3 amplitude. No significant main effects were observed for odor valence (F(2, 62) = 0.712, *p* = 0.462, η_p_^2^ = 0.024) or target type (F(1, 31) = 1.419, *p* = 0.243, η_p_^2^ = 0.047). Additionally, none of the two-way interactions odor × response direction (F(2, 62) = 0.362, *p* = 0.670, η_p_^2^ = 0.012), odor × target type (F(2, 62) = 1.885, *p* = 0.164, η_p_^2^ = 0.061), or response direction × target type (F(1, 31) = 3.887, *p* = 0.058, η_p_^2^ = 0.118) were significant. The three-way interaction among odor, response direction, and target type was also not significant (F(2, 62) = 0.350, *p* = 0.689, η_p_^2^ = 0.012). Post hoc comparisons revealed that P3 amplitude was significantly larger for avoidance responses (1.62 ± 0.32 μV) than for approach responses (0.85 ± 0.29 μV, *p* < 0.001, 95% CI [0.565, 0.972]).

**Figure 6:**
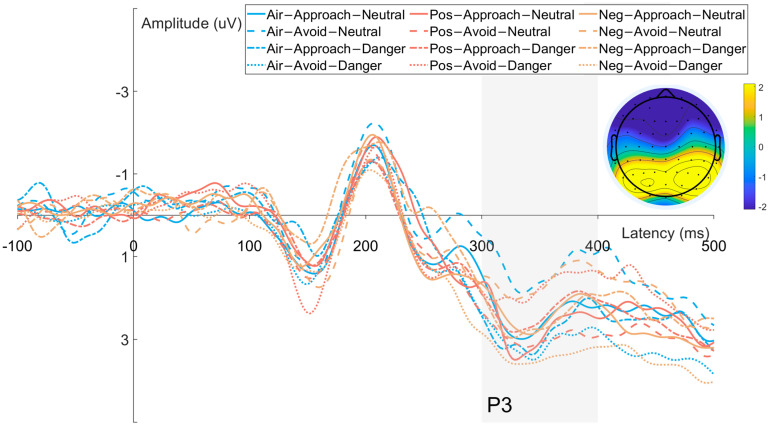
Waveforms and scalp topographies of the P3 component recorded from parietal electrode (Pz).

## 4. Discussion

This study examined how olfactory valence affects behavioral performance and the associated neural dynamics during the AAT. The core behavioral signature of approach–avoidance was stable under odor conditions, but neural data revealed clear and temporally specific modulations by olfactory cues. This ERP investigation provides novel evidence on how ambient olfactory emotional cues influence the temporal dynamics of motivated action, offering new insight into the integration of affect and motivation.

### 4.1. Behavioral Effects of Olfactory Cues on Approach–Avoidance

Consistent with previous studies on motivated behavior, participants responded faster when approaching neutral targets and avoiding dangerous targets under all odor conditions, replicating the classic behavioral feature of automatic approach-avoidance tendencies [16]. This highlights the stability of this motivational system throughout evolution.

Although odor valence exerted a main effect on reaction time, the pattern was limited: responses were slower in the air condition compared with the negative odor condition, while positive odors did not differ significantly from either. Importantly, odor valence did not interact with target type or response direction, and accuracy was unaffected by odor. These results suggest that while olfactory cues can modulate the speed of behavioral responses to some extent, they do not override the fundamental approach–avoidance tendencies triggered by visual targets.

The stability of these responses supports dual-process models of behavior, such as the Reflective-Impulsive Model [11]. In this context, the motivational response elicited by the visual target appears to serve as the dominant signal within the impulsive system, effectively overriding or filtering out the influence of ambient olfactory cues [35]. This perspective aligns with emerging models of multi-sensory integration, which propose that the hierarchy and relevance of multiple sensory inputs dictate their influence on motor output [36,37]. In our task, visually presented targets likely served as the primary drivers of the approach–avoidance response, rendering the olfactory cues secondary.

Therefore, our results highlight the relative stability of fundamental motivational behaviors under the different odor conditions. However, the lack of behavioral differences does not necessarily imply a lack of neural processing. It remains to be explored whether the brain registers and processes these olfactory signals, even if they do not translate into a measurable change in motor output.

### 4.2. A Preemptive Role for Olfactory Cues in Resolving Motivational Conflict

Our N2 findings provide evidence for a unique preemptive mechanism by which olfactory cues modulate motivational processing. Consistent with a preemptive role, both pleasant and unpleasant odors reduced N2 amplitude relative to the air condition, suggesting that olfactory input can dampen neural signatures associated with conflict monitoring before conflict is fully engaged. This reduction occurred regardless of the specific odor valence, indicating a general modulatory effect of olfactory cues on the neural processing of motivationally relevant stimuli. Moreover, N2 amplitude was larger for approach versus avoidance responses, reflecting directional differences in action-related conflict processing, but this effect did not interact with odor valence. Although we interpret the reduced N2 as reflecting preemptive state modulation, we acknowledge that alternative mechanisms, such as a reduction in reactive conflict monitoring or changes in evidence accumulation dynamics, could also contribute to the observed effect [38]. Together, these patterns suggest that olfactory cues exert a preemptive influence on neural activity related to motivational conflict, rather than altering the reactive conflict signal itself.

This distinct mechanism likely arises from olfaction’s unique neuroanatomical pathway. Unlike other sensory systems, olfactory information bypasses the thalamus and projects directly to the limbic system, including the amygdala and orbitofrontal cortex [39,40]. This direct, early access to core emotional and motivational centers allows odors to activate affective networks, which may then preemptively modulate behavior and reduce the need for reactive cognitive control [41]. This pattern differs from other emotional cognition interactions. Some studies report that emotions can influence cognitive conflict, but their influence varies depending on the type of conflict and the valence of the emotion. In some cases, emotions can even hinder conflict resolution [42,43]. Our results suggest that olfactory cues engage a more fundamental, state level modulation that reduces perceived conflict itself.

While both positive and negative odors similarly reduced the N2 effect, their underlying neural mechanisms may still differ. Research on odor perception suggests that the brain can separately process and represent the pleasant and unpleasant components of a stimulus [44]. The orbitofrontal cortex, a key region for representing reward value, plays a central role in this process [45]. Therefore, we speculate that positive odors may activate reward related circuits, which prime approach tendencies and reduce perceived conflict [46,47], whereas negative odors could activate threat-detection systems, such as the amygdala, facilitating avoidance [48]. While our study does not directly test these pathways, this model provides a framework for future research to investigate how distinct neural systems contribute to preemptive emotional modulation of executive functions.

### 4.3. A Temporal Cascade of Olfactory Influence

Our ERP findings show that olfactory cues influence motivational behavior at multiple processing stages. This temporal cascade extended from early sensory attentional responses to late decision-making processes.

The P1 component was not significantly modulated by odor valence, target type, or response direction. This indicates that very early sensory-attentional processing was largely unaffected by olfactory cues, suggesting that initial visual encoding of motivationally relevant stimuli occurs independently of ambient odors.

Under positive The N1 component showed a robust influence of both odor valence and response direction. Avoidance responses produced greater amplitudes than approach responses across odor conditions. Additionally, a significant interaction between response direction and target type indicated that early sensory attention is sensitive to the combination of action and stimulus valence, with larger amplitudes for avoidance of neutral and dangerous targets in line with their respective behavioral demands. Post hoc comparisons revealed that N1 was reduced in the air condition relative to both positive and negative odors, consistent with an early facilitation of sensory processing by olfactory input [49].

As discussed previously, both positive and negative odors reduced the typical conflict-related N2 effect, indicating that motivational control can operate in a proactive, bottom-up manner rather than as a reactive adjustment following conflict detection [50]. This preemptive influence aligns with the limbic connectivity of the olfactory system, modulating neural activity before conflict signals are fully engaged.

Finally, the P3 component reflected the main effect of response direction under all odor conditions, with avoidance responses producing greater amplitudes than approach responses. Neither odor valence nor target type significantly modulated P3, supporting its role as a stable integrator of task-relevant information during late-stage decision-making [14].

ERP results illustrate a temporal cascade of olfactory influence on motivated behavior, beginning with early sensory facilitation (N1), extending through proactive modulation of conflict monitoring (N2), and culminating in decision-related processing (P3), while leaving the earliest visual encoding stage (P1) largely unaffected. This pattern is consistent with theories emphasizing adaptive early threat detection [51] and supports the notion that positive states can preserve selective attention to danger [52].

Taken together, these results demonstrate that olfactory cues regulate attention, conflict processing, and decision-making in a coordinated cascade, shaping motivated behavior at multiple levels. Nevertheless, several limitations point to directions for future research. Despite applying ICA preprocessing to minimize EMG, ECG, and EOG artifacts, residual facial muscle activity may still influence the interpretation of EEG results. Future studies could address this by using high-density EEG combined with more advanced artifact separation algorithms or EMG correction techniques to further improve data quality. Although intermittent odor presentation was employed to reduce adaptation, residual habituation may have affected participants’ neural responses. Future research could explore more dynamic or individualized odor delivery paradigms to better account for adaptation effects. Finally, while female participants were included, the phase of their menstrual cycle was not recorded. Future studies should systematically track menstrual cycle phases and include them as covariates to examine how hormonal fluctuations modulate olfactory cognitive interactions.

## 5. Conclusions

This study examined how odor valence modulates approach-avoidance behavior and its potential neural mechanisms. Behaviorally, when actions matched the motivational valence of the target, participants responded faster and more accurately, and this pattern was stable under odor conditions.

ERP results revealed that odor valence influenced neural processing at multiple stages. Early components (N1) were modulated by odor and response direction, indicating effects on sensory-attentional processing. Under the air condition, N2 amplitudes reflected conflict monitoring. This effect disappeared under positive and negative odor conditions, suggesting that odors influence neural conflict monitoring signals rather than altering the fundamental approach–avoidance behavior. Late-stage P3 amplitudes were sensitive to response direction, with larger amplitudes for avoidance actions, reflecting stable integration of task-relevant information.

In summary, olfactory cues shaped the temporal dynamics of neural processing without changing the basic approach–avoidance behavior. These findings indicate that odors can influence the brain’s processing of motivation related behavior at multiple stages.

## Figures and Tables

**Table 1:** Evaluation results for targets.

	Neutral	Danger	*p*-Value
Familiarity	6.83 ± 0.38	6.75 ± 0.44	0.488
Dangerous	1.46 ± 0.51	6.42 ± 0.65	<0.001
Arousal	3.58 ± 0.50	3.63 ± 0.57	0.774

## Data Availability

The data presented in this study are available on request from the corresponding author subject to privacy, ethical and legal clearance.

## Abbreviation

The following abbreviation is used in this manuscript:

AAT	Approach-Avoidance Task
